# Irradiation dose to the swallowing apparatus impacts nutritional status in head and neck cancer patients—results from the prospective randomized HEADNUT trial

**DOI:** 10.1007/s00066-023-02124-3

**Published:** 2023-08-17

**Authors:** Anna Finger, Maximilian Grohmann, Linda Krause, Andreas Krüll, Cordula Petersen, Alexander Thieme, Dirk Rades, Anastassia Löser

**Affiliations:** 1 Outpatient Center of the UKE GmbH, Department of Radiotherapy and Radiation Oncology, University Medical Center Hamburg-Eppendorf, Martinistraße 52, 20246 Hamburg, Germany; 2 Department of Radiotherapy and Radiation Oncology, University Medical Center Hamburg-Eppendorf, Martinistraße 52, 20246 Hamburg, Germany; 3 Institute of Medical Biometry and Epidemiology, University Medical Center Hamburg-Eppendorf, Martinistraße 52, 20246 Hamburg, Germany; 4 Department of Medicine, Department of Biomedical Data Science, Stanford University, 1265 Welch Road, 94305 Stanford, CA USA; 5 Department of Radiotherapy, University Medical Center Schleswig-Holstein/Lübeck, Ratzeburger Allee 160, 23562 Lübeck, Germany

**Keywords:** Swallowing apparatus, Head and neck cancer, Malnutrition, HEADNUT trial, Fat-free mass index

## Abstract

**Purpose:**

To investigate the influence of radiation dose to the swallowing muscles on the nutritional status in patients with head and neck cancer undergoing primary or adjuvant (chemo)radiotherapy (C)RT.

**Methods:**

Between 2018 and 2020, 61 patients were prospectively randomized into the so-called HEADNUT trial (head and neck cancer patients undergoing nutritional intervention). Follow-up was continued until 2022. Contouring of the swallowing apparatus included the superior (scm), middle (mcm), and inferior constrictor muscle (icm), the cricopharyngeal muscle (cphm), and the esophageal inlet. Nutritional status was assessed by bioelectrical impedance analysis (BIA) at the beginning and the end of radiotherapy. The posttherapeutic nutritional status was evaluated by the BIA-derived fat-free mass index (FFMI; kg/m^2^). Malnutrition was assumed at FFMI values of < 15 (women) and < 17 (men) kg/m^2^. To find differences between dosimetric parameters in well- and malnourished patients, Mann–Whitney U test was used. To model the association between malnutrition and its potentially influencing variables, several logistic regression models were built.

**Results:**

The following parameters differed between well- and malnourished patients at the end of therapy: icm Dmean, V40Gy (%), V50Gy (%), and V60Gy (%), and sphm V40Gy (%). After entering these parameters into a multivariable logistic regression model (dosimetric model), icm Dmean (b = −0.12; Exp(b) = 0.88; 95% CI: 0.78–1.0; *p* = 0.06) and icm V40Gy (%; b = 0.06; Exp(b) = 1.07; 95% CI: 1–1.13; *p* = 0.04) proved to be independent dosimetric predictors of malnutrition. We only determined the cut-off value for predicting malnutrition for icm V40Gy (%) since it was the only parameter which met *p* < 0.05. The optimal cut-off value for the predictor V40Gy (%) based on the Youden Index was 85.6%. Another logistic regression model (dosimetric-clinical model) consisted of icm V40 (%) and the clinical parameters tumor localization, malnutrition before RT, gender, and combined chemotherapy. It was confirmed that both icm V40% (b = −1.9; Exp(b) = −2.7; 95% CI: 0.01–0.8; *p* = 0.03) and malnutrition at baseline (b = −1.9; Exp(b) = 4.4; 95% CI: 8.4–816.6; *p* = 0.0002) were independent predictors of subsequent malnutrition the end of RT.

**Conclusion:**

Establishment of a normal nutritional status before the start of RT and adherence to dose constraints for the swallowing apparatus may prevent malnutrition in head and neck cancer patients at the end of therapy. Specifically, we suggest an icm V40Gy (%) of more than 86% to be predictive for nutritional complications.

## Introduction

Nutritional status is considered to be an oncologic predictor of survival in head and neck cancer (HNC) patients undergoing radiotherapy (RT) [1–3]. Especially the combination of therapeutic modalities, e.g., chemoradiotherapy (CRT), is associated with a higher incidence of side effects like dysphagia or mucositis [4]. Consequently, a deterioration of nutritional status is preprogrammed: At treatment start, malnutrition is already present in 3–52% of patients and this rises to more than 80% during ongoing therapy [5, 6]. In addition to various anthropometric methods (e.g., weight and body mass index), bioelectrical impedance analysis (BIA) represents a scientifically valid, cost-effective, and noninvasive method for assessing nutritional status. Two BIA-derived parameters, namely phase angle and fat-free mass (FFM) index (FFMI), are good measures of nutritional status [2, 7]. The phase angle is defined by weight, height, muscle mass and fat tissue [8, 9], and suits as predictor for survival in HNC patients [10]. In 60–70% of cases, weight loss is based on a decrease in FFM [11, 12], which is reflected in the FFMI. For FFMI, threshold values for malnutrition were proposed at < 15 kg/m^2^ and < 17 kg/m^2^ in women and men, respectively [13].

Five muscular structures are relevant for swallowing: superior constrictor muscle (scm), the middle constrictor muscle (mcm), the inferior constrictor muscle (icm), the cricopharyngeal muscle (cphm), and 1 cm of the cervical esophageal inlet (eim) [14]. Being crucial due to its elevation during swallowing, the larynx constitutes another risk structure during RT [15]. The mean dose (Dmean) to the larynx (and to the icm) predicts long-term swallowing complications and percutaneous endoscopic gastrostomy (PEG) tube dependence [16–18]. Regardless of modern RT techniques like intensity-modulated radiotherapy (IMRT) or volumetric arc technique (VMAT), strict attention to the dose constraints of these risk structures is inevitable. Caudell et al. reported a Dmean of more than 41 Gy to the larynx and a laryngeal volume of more than 24% receiving 60 Gy (V60Gy > 24%) as being associated with feeding tube dependence and aspiration. For the icm, they found V60Gy > 12% to be the threshold dose for these complications. When considering the scm and mcm, a dose of 65 Gy to more than 33% (scm) and to more than 75% (mcm) of the structure’s volume was related to dilation-worthy strictures [17].

We have published our results about the impact of RT dose on feeding tube dependence in the HEADNUT study population previously [18]. In contrast, this work focuses on the influence of dose exposure of the swallowing apparatus on malnutrition in the same population.

## Materials and methods

### Study design, patient selection, and treatment

Our presented data were prospectively obtained as part of the so-called HEADNUT trial (head and neck cancer patients under [chemo]radiotherapy undergoing nutritional intervention; Clinical Trials Register: DRKS00016862) at the University Medical Center Hamburg-Eppendorf, Germany, between 2018 and 2022. This trial was approved by the local ethics committee (PV5818) and written informed consent was signed by all patients before study entry. Detailed information on study design, patient and primary and secondary endpoint selection have already been published elsewhere [3]. Briefly, the HEADNUT trial is a single-center prospective nutritional intervention study in which patients were randomized 1:1 into an intervention (with nutritional counseling) versus control (without nutritional counseling) group. All patients received BIA measurements at 2‑week intervals during ongoing RT. Only adult patients with histologically confirmed squamous cell carcinoma of the head and neck were included and treated with RT or combined CRT. A curative therapy indication and a Karnofsky performance status of at least 60% were mandatory for study participation. Patients with a second malignancy less than 15 years ago and those with an inlying pacemaker (relative contraindication for BIA) were excluded. Since we did not observe significant differences with regard to primary and secondary endpoints between the study groups during our previous analyses, all patients are considered as one collective in the present analysis [3].

IMRT was administered with single fractions of 1.7–2.0 Gy, 5 times/week, to total doses varying from 60–70.4 Gy alone or as combined CRT with mainly cisplatin (100 mg/m^2^ every 3 weeks or 40 mg/m^2^ weekly) [19, 20]. In case of contraindications, 5‑fluorouracil (5-FU) and mitomycin C (MMC; 600 mg/m^2^ on days 1–5 and 10 mg/m^2^ on days 5 and 36, respectively) were given [3].

### Contouring of the swallowing apparatus

During prospective treatment planning, contouring of the swallowing muscles (as one structure) and the larynx was performed on 3‑mm axial planning CT slices (Somatom, Siemens Healthcare, Forchheim, Germany) using Eclipse (v15.1, Varian Medical Systems, Inc., Palo Alto, CA, USA). For a more precise distinction, the substructures of the swallowing musculature (scm, mcm, icm, cphm, and eim) were retrospectively contoured by the same physician. Anatomical organ delineation was performed in accordance with Levendag et al. (scm: mid C2 to upper C3; mcm: upper C3 to upper C4/caudal part of the corpus of the hyoid bone; icm: upper C4 to mid C6; cphm: mid C6 to esophageal junction) [14, 18].

Besides the gross target volume (GTV), the following dose characteristics were extracted for all substructures of the swallowing musculature and the larynx: Dmax (maximum dose), Dmean (in Gy), V40Gy (organ volume (%) that received ≥ x Gy), V50Gy, V60Gy, and V65Gy (in %).

### Statistics

Nonnormally distributed data were expressed by the median (with the corresponding range) and normally distributed data by the mean (± standard deviation, SD).

For univariable analysis, Mann–Whitey U test was applied to compare whether two independent groups are different. For comparisons within cross tabulations of two categorical variables with only two levels each, Fisher’s exact test was used. To examine relationships between variables that are either nominally or ordinally scaled with more than two expressions, chi-square test was applied.

To model the association between malnutrition and its potentially influencing variables, four different logistic regression analyses were applied:

For the first logistic regression model, all DVH parameters showing a *p* < 0.05 were entered into two different multivariable logistic regression models (one model for only laryngeal DVH and a second model for muscular structures of the swallowing apparatus). Based on the *p*-value, no parameters emerged from the first laryngeal model that could be identified as influential variables, whereas five potential influencing DVHs emerged from the second muscular model. To exclude potential covariable interactions, these five DVH parameters were then re-investigated for their predictive performance in a separate (third) logistic model.

Our fourth logistic regression model (dosimetric clinical model) consisted of the resulting parameter still showing *p* < 0.05, i.e., icm V40 (%), and other clinically relevant parameters (tumor localization, malnutrition before RT, combined chemotherapy, and gender).

To define cut-off values, the Youden index was derived from receiver operating characteristics (ROC).

For multivariable logistic regression analysis and ROC with Youden index calculation, MedCalc (version 19.6, MedCalc Software Ltd, Ostend, Belgium) was used. All other calculations were performed with SPSS (version 28.0, IBM Corp., Armonk, NY, USA).

All *p*-values in this exploratory study are used as descriptive measures. No adjustment for multiple testing was performed.

## Results

### Patient characteristics

For this analysis, complete patient data were available in 60 patients. Basic patient characteristics (together with a CONSORT chart) have already been published elsewhere [3, 18].

Pretreatment dysphagia was known in 19 patients (31.7%), with 8 in the definitive and 11 patients in the adjuvant treatment setting [18]. During the entire follow-up period, 2 patients reported their individual most severe grade of dysphagia already before the start of RT. Both patients had undergone prior surgery and now presented for adjuvant (C)RT. As expected, all patients suffered from various degrees of dysphagia under (C)RT, including 25 patients with at least dysphagia grade III (41.7%; Table 1). Also, the most severe individual dysphagia grade was reported within the time interval of ongoing radiation (especially end of [C]RT) by 57 patients (95%). In one patient, the exact timepoint of the worst dysphagia manifestation was not definable.

**Table 1:** Summary of patient characteristics in addition to previously published data [3, 18]

	Nutritional status		*p*-value
	Normal (*n* = 46)	Poor (*n* = 14)	
*Age (years)*	63 (±11.8)	64 (±10.1)	0.88^a^
*Gender*			
Male	34 (73.9%)	9 (64.3%)	0.51^b^
Female	12 (26.1%)	5 (35.7%)	
*Presence of dysphagia during RT*			
≥ CTCAE grade III (*n*)	21 (45.7%)	4 (28.6%)	0.36^b^
*Presence of dysphagia during 1st follow-up*			
≥ CTCAE grade III (*n*)	3 (6.5%)	2 (14.3%)	0.15^b^
*Presence of dysphagia during 2nd follow-up*			
≥ CTCAE grade III (*n*)	4 (8.7%)	0	0.47^b^
*Presence of nausea (at therapy completion)*	8 (17.4%)	5 (35.7%)	0.27^b^
*∆ BMI (kg/m*^*2*^*)*	−1.2 (±1.5)	−0.4 (±1.3)	0.08^a^
*∆ Phase angle (°)*	−0.6 (±1.2)	0.5 (±1.2)	0.004^a^

For this analysis, complete patient data were available in 60 patients. Poor nutritional status/malnutrition at therapy completion was defined at a FFMI < 15 (♀) and < 17 (♂) kg/m^2^. A mean (± standard deviation) is shown when data approximately follow a normal distribution. First follow-up took place 6–8 weeks after treatment completion. Second follow-up took place at least half a year after the first follow-up

**∆ **BMI (kg/m^2^) and ∆ Phase angle (°) denote the differences between the corresponding final value at therapy completion from the baseline value

*RT* radiotherapy; *CTCAE* Common Toxicity Criteria of Adverse Events; *BMI* body mass index; *FFMI* fat-free mass index

^a^Mann–Whitney U test

^b^Fisher’s exact test

At first follow-up (6–8 weeks after RT completion), 27 patients suffered from dysphagia (without differences between the well- and malnourished cohort; *p* = 0.3). Three patients had passed away (one death each was tumor- and nontumor-associated, and in the third patient the cause of death remained unknown) before the first follow-up date. The number of patients suffering from ≥ CTCAE grade III dysphagia fell to 5 patients (Table 1).

The second follow-up exam (at least 6 months later) showed 26 patients suffering from grade I–III dysphagia without differences between the well- and malnourished group (*p* = 0.81). Four more patients had died before the second follow-up exam (two due to tumor-related causes).

Additional patient characteristics are summarized in Table 1. Normally and malnourished patients only differed in terms of changes in the post- to pretherapeutic phase angle (∆ phase angle): ∆ phase angle between these two timepoints was more pronounced in patients with a good nutritional status at therapy completion (*p* = 0.004). We did not observe other differences between normally and malnourished patients in this cohort.

### Univariable analysis: DVH parameters and nutritional status

Firstly, we set out to determine differences in all of the abovementioned DVH parameters between normally nourished and malnourished patients. This univariable analysis revealed nine DVH parameters of the structures larynx, icm, and cphm which differed between patients with normal FFMI values and those presenting with malnutrition (Mann–Whitney U test). Table 2 summarizes relevant results (with *p* < 0.05).

**Table 2:** Differences in DVH parameters depending on nutritional status

	Nutritional status at therapy completion		*p*-value^a^
	Normal (*n* = 46)	Poor (*n* = 14)	
*Larynx*			
Dmean (Gy)	39 (±14.7)	51.9 (±15.6)	0.03
V50Gy (%)	11.2 (0–100)	71.7 (0.3–100)	0.02
V60Gy (%)	0.3 (0–99.8)	21.8 (0–100)	0.03
V65Gy (%)	0 (0–89.8)	4.3 (0–99.9)	0.014
*Icm*			
Dmean (Gy)	50.7 (0.2–69.8)	59.8 (32.1–69.9)	0.004
V40Gy (%)	83.3 (0–100)	100 (20.8–100)	0.006
V50Gy (%)	57 (0–100)	95.7 (12.2–100)	0.007
V60Gy (%)	13.4 (0–100)	55.1 (0.1–100)	0.02
*Cphm*			
V40Gy (%)	51 (0–100)	100 (0.2–100)	0.03

Comparison of dose parameters with *p* < 0.05 between normally nourished and malnourished patients. Poor nutritional status/malnutrition was defined at an FFMI < 15 (♀) and < 17 (♂) kg/m^2^. A mean (± standard deviation) is shown when data approximately follow a normal distribution, otherwise the median (range) is shown

*DVH* dose–volume histogram*; FFMI* fat-free mass index; *icm* inferior constrictor muscle; *cphm* cricopharyngeal muscle; *Dmean* mean dose; *VxGy (%)* organ volume (%) that received ≥ x Gy

^a^Mann–Whitney U test

### Multivariable analysis

To avoid model overfitting, four different logistic regression models were established. The first model consisted of the laryngeal substructures from univariable analysis (dosimetric laryngeal model) with *p* < 0.05 (Dmean [Gy] V50Gy [%], V60Gy [%], V65Gy [%]). However, no laryngeal parameters remained within this multivariable logistic regression model, meaning that there was no variable with *p* < 0.05 left after running the multivariable model.

**Fig. 1:**
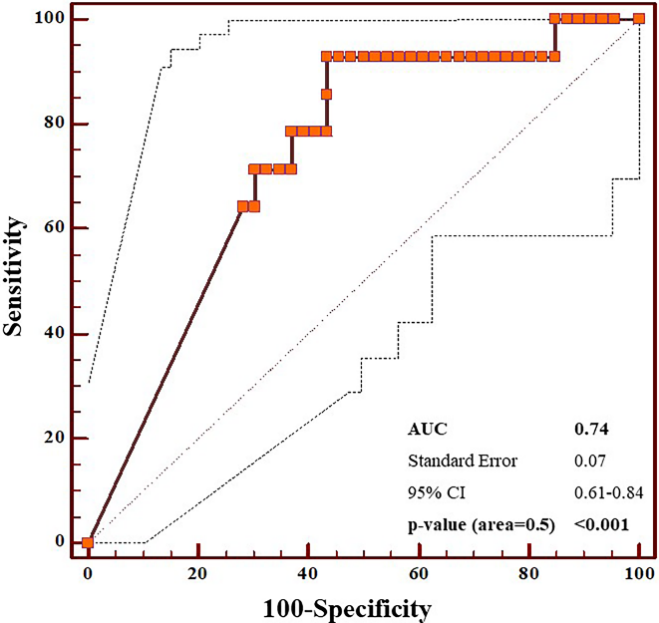
Plot of ROC analysis for icmV40Gy (%) indicating the 95% interval (*dashed line* on both sides of the curve). Youden index corresponded to 0.49 with the associated criterion of > 85.63%. *Icm* inferior constrictor muscle, *ROC* receiver operating characteristics, *AUC* area under the curve; *95% CI* 95% confidence interval

Our second model exclusively contained the muscular structures of the swallowing apparatus with *p* < 0.05 from univariable analysis (dosimetric muscular model). Icm Dmean (Gy), V40Gy (%), V50Gy (%), and V60Gy (%), and cphm V40Gy (%) remained within this model. To exclude interrelation of variables, another third model (consisting of these five parameters) was calculated. Icm Dmean (b = −0.12; Exp(b) = 0.88; 95% CI: 0.78–1.0; *p* = 0.06) and icm V40Gy (%; b = 0.06; Exp(b) = 1.07; 95% CI: 1–1.13; *p* = 0.04) proved to be independent predictors of malnutrition. However, we only determined the cut-off value for icm V40Gy (%), since this was the only parameter which met *p* < 0.05. After performing an ROC analysis with Youden index calculation, 85.6% resulted as cut-off value (see Fig. 1).

Since not only dosimetric but also clinical parameters may influence nutritional status, a fourth model (dosimetric clinical model) consisting of icm V40 (%) and other clinically relevant parameters (tumor localization, malnutrition before RT, combined chemotherapy, and gender) was calculated. It was confirmed that both icm V40% (b = −1.9; Exp(b) = −2.7; 95% CI: 0.01–0.8; *p* = 0.03) and malnutrition at baseline (b = −1.9; Exp(b) = 4.4; 95% CI: 8.4–816.6; *p* = 0.0002) were independent predictors of subsequent malnutrition the end of RT (overall significance level of the model: *p* < 0.0001).

## Discussion

Overall, this prospective nutritional intervention study identified icm V40Gy (%) as a relevant parameter predictive of malnutrition at the end of RT or combined CRT. Regardless of our intensive literature research, we did not detect any publications relating doses to the swallowing apparatus to malnutrition (as defined by BIA). Therefore, we extended our research to an indirect surrogate parameter of malnutrition, namely known dysphagia or other swallowing complications.

To identify dosimetric predictors of long-term swallowing complications, Caudell et al. investigated 83 patients undergoing definitive RT due to squamous cell HNC [17]. PEG tube dependence at 12 months, aspiration, or pharyngoesophageal strictures served as indirect measures of long-term dysphagia. These authors concluded that the doses to the larynx and pharyngeal constrictors were related to long-term dysphagia [17]. In accordance, Caglar et al. reported that the volume of the larynx and icm receiving at least 50 Gy was associated with an increased risk of aspiration and strictures after IMRT [16]. In our previous analysis, we also found that laryngeal dose exposure (specifically V50Gy ≥ 53%) was associated with long-term PEG dependence [18]. However, the present analysis did not confirm the relationship between laryngeal dose and malnutrition. Indeed, after conducting univariable analysis, several laryngeal dosimetric parameters were identified as possible influencing factors of malnutrition. However, this was not confirmed in the subsequent multivariable logistic regression analysis, implying that they were not independent predictors when combined in one model. After this finding, we recalculated whether malnutrition at the end of therapy was associated with PEG dependence after 6 and after 12 months. An association could not be proven (*p* = 0.26 and *p* = 0.21, respectively). The approach of Rutter et al. could serve as an explanation for this finding: although patients with long-term tube dependence often suffer from dysphagia, these authors have observed that early tube placement may prevent weight loss in HNC patients under definitive RCT [21]. Whether this effect of preventing weight loss can automatically be transferred to the time after the end of RT remains unclear.

Besides the glottic and supraglottic larynx and esophagus, Feng et al. proposed the pharyngeal constrictors to be associated with swallowing complications when mean doses of > 60 Gy or a volume receiving 65 Gy of more than 50% were exceeded [22]. In comparison, Caudell et al. described a threshold value for icm V60Gy at 12%, while other authors suggested V60Gy > 60% to be connected to increased dysphagia [17, 23]. In this present analysis, icm V40Gy (%) exceeding 86% was a negative influencing variable for nutritional status.

The main limitation of this analysis is its small sample size, which implies a reduced power to detect small effects. To avoid model overfitting, four separate multivariable logistic regression models were built. At the same time, it cannot be determined to what extent the variables would have influenced each other across models. However, it must be emphasized that not only dosimetric factors exist that influence subsequent malnutrition, but that also diverse clinical variables may have an impact. To avoid model overfitting, we tested only four additional variables (combined chemotherapy, gender, malnutrition at baseline, and tumor location). Certainly, the question of the use of an inlying gastrostomy tube under ongoing RT could also have been an interesting parameter. Since, in our experience, it cannot automatically be assumed that patients regularly used the feeding tube despite its insertion, we decided not to include this parameter. Furthermore, the variables tested here represent only a selection of possible influencing variables.

The initial study design included two study groups, namely an intervention group and a control group. However, it has already been shown in our previously published work [3] that both groups are similar. Therefore, all patients were considered as one large study population for the present analysis [3, 24]. A psychological bias cannot be ruled out in multiprofessional care by a permanent study team [3, 18]. In addition to general supportive measures including appropriate analgesia (in case of odynophagia) and/or (prophylactic or reactive) gastrostomy tube placement in cases where adequate oral nutritional intake was no longer possible, the corresponding nutritional support was highly individualized and implemented accordingly by our multiprofessional study team depending on the possibilities of the nutritional form (oral nutritional intake versus gastrostomy tube). Accordingly, variation was very limited in patients fed exclusively by an inset feeding tube. This may complicate interindividual patient comparisons.

In conclusion, malnutrition at the end of therapy was strongly associated with malnutrition at baseline and the dose exposure to icm V40Gy (%), emphasizing the need to maintain a good nutritional status even before RT start and to undercut corresponding dose constraints.
